# Human Micro-Expressions in Multimodal Social Behavioral Biometrics

**DOI:** 10.3390/s23198197

**Published:** 2023-09-30

**Authors:** Zaman Wahid, A. S. M. Hossain Bari, Marina Gavrilova

**Affiliations:** Biometric Technologies Laboratory, Department of Computer Science, University of Calgary, 2500 University Dr. NW, Calgary, AB T2N 1N4, Canada

**Keywords:** natural language processing, social behavioral biometrics, biometric identification, person identification, human micro-expression

## Abstract

The advent of Social Behavioral Biometrics (SBB) in the realm of person identification has underscored the importance of understanding unique patterns of social interactions and communication. This paper introduces a novel multimodal SBB system that integrates human micro-expressions from text, an emerging biometric trait, with other established SBB traits in order to enhance online user identification performance. Including human micro-expression, the proposed method extracts five other original SBB traits for a comprehensive representation of the social behavioral characteristics of an individual. Upon finding the independent person identification score by every SBB trait, a rank-level fusion that leverages the weighted Borda count is employed to fuse the scores from all the traits, obtaining the final identification score. The proposed method is evaluated on a benchmark dataset of 250 Twitter users, and the results indicate that the incorporation of human micro-expression with existing SBB traits can substantially boost the overall online user identification performance, with an accuracy of 73.87% and a recall score of 74%. Furthermore, the proposed method outperforms the state-of-the-art SBB systems.

## 1. Introduction

Biometric methodologies are recognized for their ability to confirm an individual’s identity by analyzing unique physical or behavioral attributes [1,2]. In the realm of physiological biometrics, the physical characteristics of a person are coded to form a distinctive profile that can later be used for identification [3]. This category of biometrics frequently incorporates fingerprint, iris, palm, face, and hand geometry analysis [1,3]. In contrast, behavioral biometrics focus on an individual’s unique patterns of behavior, including distinct ways of speaking, walking, typing, and signature writing [4,5].

Emerging from the rapid expansion of social media, Social Behavioral Biometrics (SBB) is a novel biometric category. This innovative field of study investigates a person’s social interactions and communication patterns to ascertain their identity [6]. Given the dramatic growth in social media users, Online Social Network (OSN) platforms have become data-rich environments, providing valuable insights into user behaviors. With this wealth of data, SBB has found successful applications across a range of fields, such as person authentication, anomaly detection, behavioral analysis, risk assessment, and situation awareness [6,7,8].

A burgeoning area of research within the field of social behavioral biometrics is the utilization of behavioral traits for biometric identification. Recent scholarly endeavors have delved into various aspects of social behavior, such as retweet networks, Uniform Resource Locator (URL) networks, reply networks, hashtag networks, temporal networks, linguistic profile, and human micro-expression from text as potential biometric identifiers [9,10,11]. Harnessing these social behavioral biometrics offers potential avenues for bolstering cyberspace security and surveillance [12]. Moreover, SBB traits have found diverse applications in areas such as digital banking [13], spam detection online [14,15], assessing trustworthiness in social media [16,17], identifying sexual predators [18], detecting cyberbullying [19], exploring psychological states [20], predicting mental health conditions [21], and facilitating the early detection of depression [22].

One of the latest social behavioral biometrics, human micro-expression, demonstrates the capacity for online biometric identification [11]. The concept of human micro-expressions extends from the domain of visual facial expressions to textual analysis in the state of the art. In a textual context, micro-expressions could be understood as subtle cues or indicators that suggest an individual’s emotional state or reaction [23,24]. These can be certain patterns of language, choice of words, frequency of certain phrases, and even the speed and timing of responses [23,24]. Analyzing these textual micro-expressions not only offers insightful details about an individual’s emotional state and sentiments but also reveals distinctive behavioral patterns for biometric identification [11]. However, questions remain unanswered and uninvestigated on the efficacy of human micro-expression biometric traits from individuals’ written samples for comprehensive biometric identification in a multimodal system. This paper explores and answers the following research questions:

- Can human micro-expression from text be integrated with other SBB traits for biometric identification of online social network users?
- Does the integration of human micro-expression from text to multimodal SBB systems contribute to improved biometric identification accuracy?

The following contributions are made in this paper:

- A multimodal SBB system is proposed, integrating the human micro-expression biometric with other SBB traits. The fusion of the human micro-expression biometric with additional SBB traits results in improved user recognition rates, showcasing the compatibility and significance of this novel biometric trait within multimodal frameworks.
- A rank-level fusion technique is employed based on the weighted Borda count method within the proposed multimodal SBB system architecture. The performance of the proposed method is compared with the state-of-the-art multimodal systems and demonstrated that the proposed multimodal SBB system architecture outperforms the existing ones.

The paper is organized as follows: Section 2 discusses the existing research in the domain of SBB, Section 3 delves into the details of the proposed method, Section 4 presents the experimental results and analyses, and Section 5 summarizes the study with a discussion of the potential future research.

## 2. Related Works

The concept of Social Behavioral Biometrics (SBB) was first unveiled in 2014 [6]. Subsequently, there has been a growing interest and surge in advancements within the SBB discipline. Sultana et al. [6] introduced the framework for both unimodal and multi-modal SBB systems based on user social patterns. They discussed Dunbar’s number theory [25], which suggests cognitive limits to the number of stable social connections a person can maintain, thereby providing a basis for user identification [25]. Despite the proliferation of online social networks allowing thousands of connections, studies assert that cognitive thresholds still apply [26,27], reinforcing the idea that a person’s primary circle of friends, exhibiting distinct behavioral patterns, can be used to identify them.

Later, the research in [9] introduced key SBB traits, including the reply and retweet networks. Both networks serve as social behavioral biometrics and are generated based on how often a user responds to or retweets content from their digital acquaintances [9]. They represent social interactions as network structures, with nodes being users and edges denoting interactions [9]. They differ in their data sources, with the reply network focusing on user responses and the retweet network revolving around content redistribution. Logarithmic frequency of weights is assigned to the edges in these networks to establish them [9]. Among these two, the reply network exhibited superior performance in identifying individuals.

Further, the trendy topic profile captures users’ hashtag usage behaviors to understand topic-wise interests and preferences, and the URL network maps users’ domain preferences and sharing patterns in online social networks [9]. Additionally, a temporal profile was designed to capture the temporal information regarding an individual’s online activity [28]. The temporal profile yields a wealth of data, including the average daily probability of tweeting, average hourly tweet frequency, patterns of tweeting across seven-day intervals, specific times of tweeting throughout the week, and others, while offering statistical data-rich insights. The temporal profile demonstrates lower performance in comparison to other SBB traits [28].

Language-based identification was also explored by Sanjida et al. [10,29]. They designed linguistic profiles as an SBB trait for online user authentication to identify writing style, vocabulary, and other language attributes. A Term Frequency-Inverse Document Frequency (TF-IDF) technique was utilized to extract the linguistic profiles of OSN users. The method was validated with machine learning classifiers, demonstrating a high degree of accuracy in recognizing users through linguistic attributes [10].

SBB features have been applied to enhance online safety lately. A sexual predator detection system is introduced in [18] that extracts SBB features from textual conversations to build user profiles, and by focusing on specific features, the system was able to detect potential threats with high accuracy. In [30], an innovative method was proposed to improve both personalized services and the security of Internet of Things (IoT) devices by leveraging SBB features. By incorporating continuous verification intelligence into smart devices, a high user verification rate was reported. In addition, personality traits were explored as a form of SBB features to evaluate user trustworthiness online [16], demonstrating the potential of SBB traits in fostering trust in online interactions. Lastly, Saleema and Thampi [31] put forth an innovative approach to user identification in online social networks, using behavior modeling based on cognitive psychology. This approach amalgamated user-generated content with social interaction data, taking into account aspects like memory, learning, and perception, thereby achieving a comprehensive understanding of user behavior [31]. The model was able to discern between authentic and fake users, indicating the potential of cognitive psychology-based behavior modeling for user identification in online social networks [31].

In the latest advancement of SBB, Zaman et al. [11] introduced human micro-expression as a novel social behavioral biometric. This method transforms users’ microblogs into emotion signals by leveraging Parrott’s [32] six primary emotions [11]. An emotion detection model was developed to extract these six fundamental emotions from the user-generated content [11]. Later, the emotion signals and their corresponding mappings are constructed using an original technique proposed in [11]. Dynamic Time Warping (DTW) algorithm [33] was subsequently employed to extract emotion-progression features within pairs of emotion signals, leading to the creation of a comprehensive human micro-expression profile for users on online social networks. A rank-level fusion with weighted Borda count technique [34] is used to generate the final decision score for user identification. The identification of users based on solely human micro-expression biometrics demonstrated promising results.

One of the key limitations of the recent SBB trait is that while the standalone identification performance is reasonably good, the identification process is not comprehensive, resulting in underperformance when compared to existing multimodal SBB systems. Furthermore, the compatibility of human micro-expression for comprehensive user identification by looking at several SBB traits is still unknown. Therefore, the incorporation of human micro-expression not only reveals the integration capacity of the emotion-based SBB features with the network, temporal, and linguistic-based SBB features but also demonstrates the potential ability to improve the existing identification performance in a multimodal framework. This aspect necessitates further research to bridge the gap between individual SBB traits and a comprehensive, multimodal user identification approach.

## 3. Methodology

This section provides a detailed description of the proposed method for biometric identification of users. The system is designed to identify users based on their social behavioral biometrics, which are extracted from their online social network profiles. The system is comprised of four key components: data preprocessing, SBB trait generation, matching score generation, and score fusion and identification.

### 3.1. Overview

**Figure 1:**
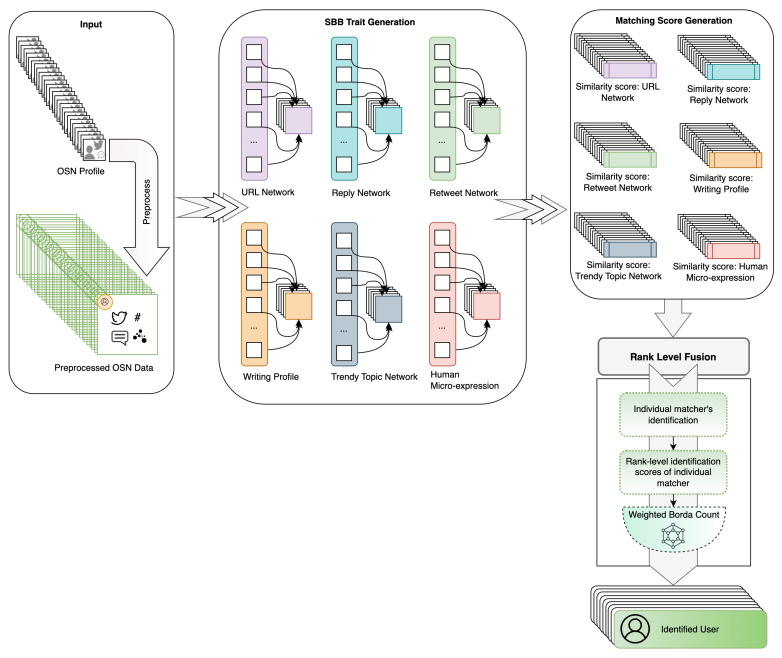
Workflow of the proposed method for multimodal SBB system.

The approach proposed for comprehensive biometric user identification hinges on the analysis of social behavioral traits and is depicted in Figure 1.

The initial input to the proposed method is the online social network (OSN) profile of a user. This profile, comprising a variety of user data, undergoes an initial preprocessing stage, where the crucial pieces of information that hold relevance to the identification process are extracted. The data is then passed on to the SBB trait generation stage, where the data is transformed into a set of SBB traits. These traits, constituting the essence of the user’s social behavior, provide the fundamental data upon which the identification process hinges. Moving forward, the extracted SBB traits are then used to generate matching scores, which are subsequently fused to produce a final decision score. For score fusion, a rank-level Weighted Borda Count [34] technique is applied to the matching scores produced by each SBB trait. The final decision score is then used to identify the user. The details of each of these stages are discussed in the following sections.

### 3.2. Data Preprocessing

In most SBB research, Twitter [35] is the most frequently used OSN for SBB trait extraction. Given Twitter’s inherent informality and unstructured nature, it is crucial to exercise additional care and implement appropriate measures when handling its data [36]. To generate various SBB traits, diverse data modalities must be preserved and preprocessed accordingly. The following process is employed to preprocess the OSN profile data of users effectively.

#### 3.2.1. Filtering Relevant Data

Within an OSN profile, various types of data exist, not all of which are relevant or utilized for generating state-of-the-art SBB traits. Consequently, it is essential to identify and remove such extraneous data from the OSN profiles to streamline the analysis process and focus on the most pertinent information for generating accurate and meaningful SBB traits. Therefore, the data in the OSN profile that do not contribute to the generation of SBB traits are removed.

#### 3.2.2. Data Cleaning and Preprocessing of Textual Data

In the process of creating SBB traits, noise removal, and data cleaning are first undertaken on the filtered data to eliminate duplicates, irrelevant symbols, and data inconsistencies, ensuring each entry uniquely represents user behavior [37,38]. Following this, the textual data undergo preprocessing using standard NLP techniques, such as tokenization, normalization, stopword removal, and lemmatization, to prepare it for machine learning models designed to learn users’ behavioral traits [38,39,40].

#### 3.2.3. Identifying and Preprocessing of Network Data

Another vital aspect involves identifying various data types within the OSN profile that capture the intricate network of interactions between OSN users. This information is pivotal in understanding the social dynamics, behavioral patterns, and relationships among OSN users, ultimately providing valuable insights into their online activity, preferences, and influence within the network [39,41]. In this step, the hashtag information is filtered, which contributes to the development of a trendy topic network; the URLs are filtered and preprocessed to understand the OSN users’ most preferred domains. Most importantly, the other OSN users with whom an OSN user most frequently interacts through mentions or sharing their contents are categorized for other SBB traits.

### 3.3. Generation of SBB Traits

In this phase, various SBB characteristics are derived from the preprocessed OSN data, enhancing the overall SBB system’s effectiveness. The focus is on extracting six key SBB traits, including human micro-expression [11], writing profile [10], reply network [9], retweet network [9], URL network [9], and trendy topic network [9]. Although the temporal profile [28] is an additional SBB trait found in contemporary research, it is deliberately excluded from the proposed multimodal SBB system. This decision stems from the subpar standalone performance of the temporal profile, which does not significantly contribute to the improvement of the overall SBB system, in comparison to other SBB traits. The process of generating the six state-of-the-art SBB traits is described below.

#### 3.3.1. Human Micro-Expression

**Figure 2:**
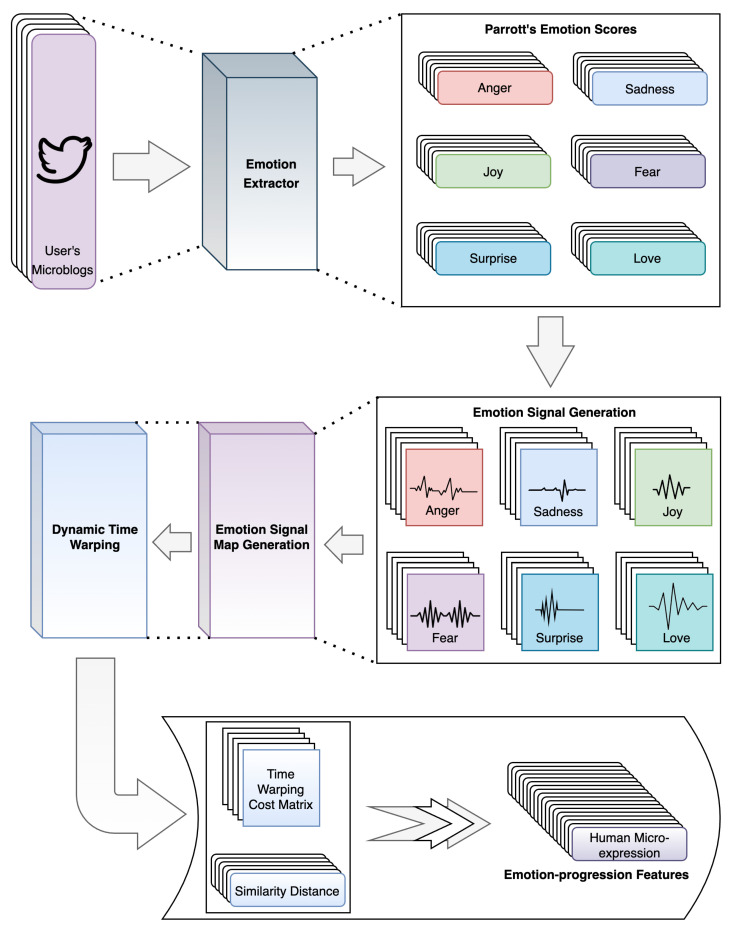
Process of generating human micro-expression SBB trait, adapted from [11].

The novel human micro-expression SBB trait [11] extracts the unique emotion-progression patterns of OSN users from their written microblogs for effective online identification. This innovative approach transforms microblogs into a series of emotion signals that subsequently form the basis for emotion-progression patterns. This generation process is visually depicted in Figure 2.

**Figure 3:**
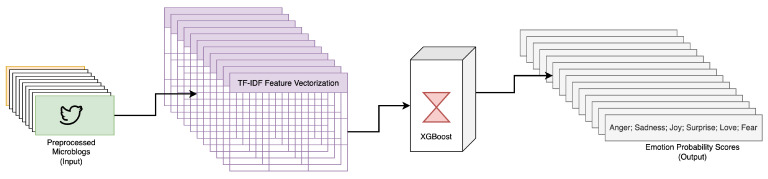
Workflow of extracting Parrott’s six primary emotions, adapted from [11].

An integral part of the procedure involves an emotion detector that employs the Term Frequency–Inverse Document Frequency (TF-IDF) vectorization technique [42]. The detector converts the microblogs into a numerical vector, which is subsequently analyzed by an XGBoost model [43] to predict the respective emotion probability scores, depicted in Figure 3.

Following this, a novel algorithm described in [11] constructs emotion signals and corresponding emotion-signal maps for each of Parrott’s primary emotions. In particular, the emotion scores of a sequence of 50 microblogs with their temporal orders are considered to construct a single emotion signal [11]. Essentially, these emotion signals serve as representations of the sequence of emotion probability scores for each OSN user’s microblogs [11]. Correspondingly, emotion-signal maps denote pairs of emotion signals, purposed for similarity feature extraction. Once the emotion signals and their corresponding emotion-signal maps are constructed, the Dynamic Time Warping (DTW) algorithm [33] is employed to extract the similarity features from the emotion-signal maps. The similarity features, time-warping cost matrix, and similarity distance, then collectively form the human micro-expression SBB trait unique to each OSN user [11].

#### 3.3.2. Writing Profile

**Figure 4:**
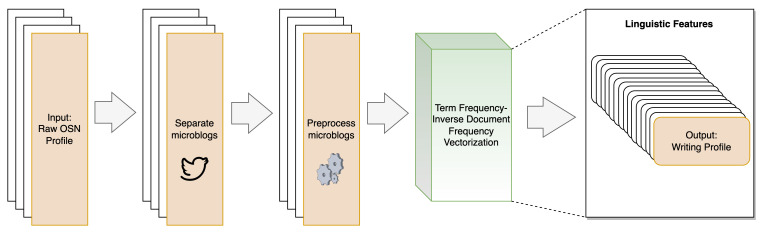
Process of generating Writing Profile SBB trait, adapted from [10,44].

The Writing Profile SBB trait, as presented in [10], explores a user’s linguistic patterns, including word choice, grammar, and sentence structure, to discern distinctive writing signatures [10,44]. Figure 4 depicts the workflow of extracting the linguistic features.

Initially, the microblogs are separated from the raw OSN profile data. To enable more effective and meaningful linguistic feature extraction, these microblogs undergo preprocessing using techniques such as tokenization, noise removal, stopwords elimination, and stemming [38,40]. Following the preprocessing, the Term Frequency–Inverse Document Frequency vectorization technique is employed to extract linguistic features within the microblogs authored by OSN users. This method identifies the unique writing patterns of OSN users based on their vocabulary choices [10,44]. These numerical features suitable for machine learning model training are the writing profiles of OSN users.

#### 3.3.3. Reply Network

**Figure 5:**
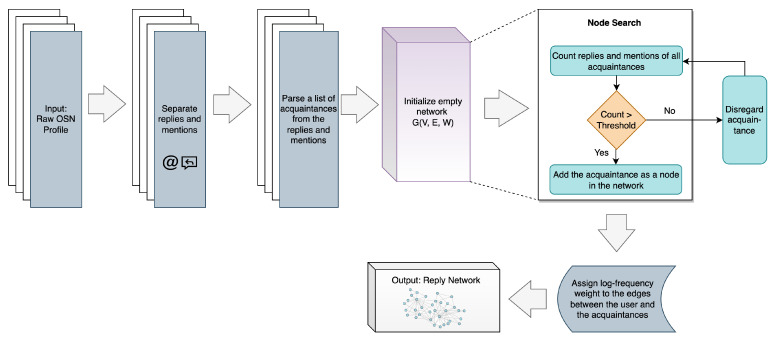
Process of generating Reply Network SBB trait, adapted from [9,44].

The Reply Network, initially introduced in [9], is a pioneering SBB trait that detects statistical patterns in the frequency of OSN users’ responses to their virtual acquaintances, in the form of replies and mentions [9]. The process of reply network generation is depicted in Figure 5.

From the raw OSN profile, data pertaining to replies and mentions are extracted and subsequently analyzed to generate a list of acquaintances. This information is then used to create a graph network consisting of nodes and edges, with each unique acquaintance represented as a node, and the OSN user serving as the edge [9,44]. To establish the set of nodes and edges, the algorithm counts the replies and mentions for each acquaintance and assesses whether a specific threshold has been reached. If the threshold is satisfied, the corresponding acquaintance is incorporated into the network as a node. Edges are formed between nodes based on the reply and mention relationships, and weights are allocated to these edges according to the frequency of mentions and replies among the nodes [9,44]. For example, an edge connecting two users will be assigned a higher weight if both users frequently communicate with each other, whereas a lower weight will be attributed in the absence of such frequent communication [9,44]. Given a term frequency (TF) of edge *t* in profile *d*, the weights are determined using the following Equation (1) [45]:

(1) $$W_{t}=1+\log\left({\mathrm{TF}}_{t,d}\right)$$

where $W_{t}$ is the log-frequency weight of edge *t*.

#### 3.3.4. Retweet Network

**Figure 6:**
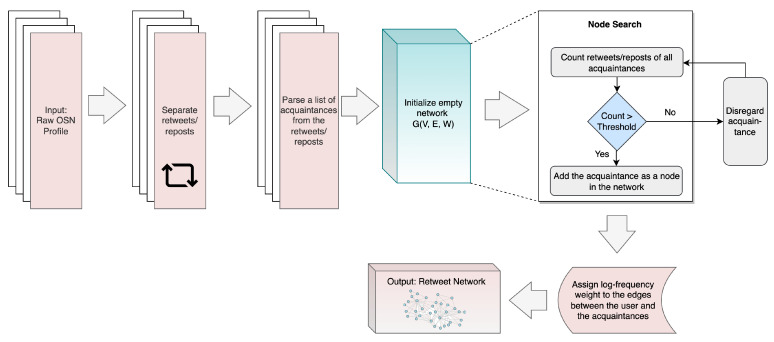
Process of generating Retweet Network SBB trait, adapted from [9,44].

The Retweet Network concept shares similarities with the Reply Network but focuses on identifying statistical patterns in the frequency of OSN users’ interactions with their virtual acquaintances through retweets or reposts [9]. Figure 6 illustrates the step-by-step process of constructing this network.

Initially, retweet and repost data are extracted from the raw OSN profile. A list of acquaintances is then derived from this data, and a weighted retweet network is established based on the retweet or repost relationship between an OSN user and their connected acquaintances. Analogous to the Reply Network, acquaintances comprise the nodes, while the user represents the edges. Nodes are created after counting the retweets for each acquaintance and determining whether a specified threshold has been met. Edge weights are assigned according to the logarithmic frequency of retweeting or reposting events between a node and an edge, with Equation (1) employed for calculating log-frequency. Finally, the algorithm generates a weighted retweet network as its output.

### 3.4. URL Network

The URL Network constructs a graph network representing an OSN user’s browsing patterns, especially the most preferred websites [9,44]. Individuals typically explore websites based on their personal interests and desire for information. The way users navigate these sites can be viewed as a unique behavioral signature for each person [9,44]. Additionally, the distinct patterns found in shared URLs can help identify users [44]. The web links they often share reflect their individual interests and preferences, which differ from one person to another, thus linking to their distinct browsing behavior as mentioned earlier [44].

**Figure 7:**
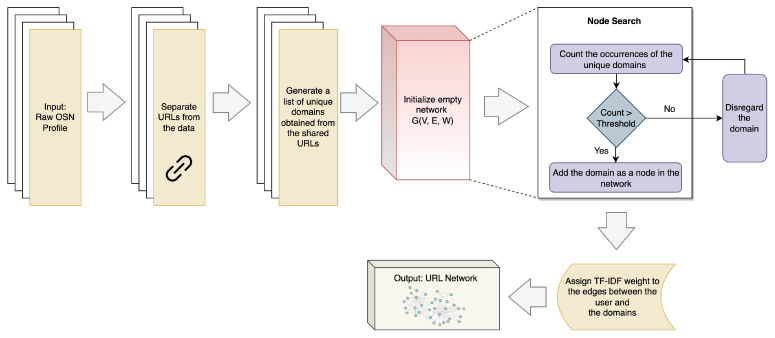
Process of generating URL network SBB trait, adapted from [9,44].

Figure 7 demonstrates the methodology for constructing the URL network for OSN users. To start, the shared URLs are extracted from the OSN profile of a user, and a list of unique domains shared by the user is compiled from these URLs. The URL network is then assembled based on the shared domains and their frequency [9,44]. In this network, each unique domain serves as a node, while the user represents the edge. A domain is added as a node only if the number of occurrences for that domain meets a predetermined threshold [9,44]. In the final step, the Term Frequency–Inverse Document Frequency (TF-IDF) vectorization technique [42] is employed to calculate the weights of the edges, resulting in the completed URL network [9,44].

### 3.5. Trendy Topic Network

**Figure 8:**
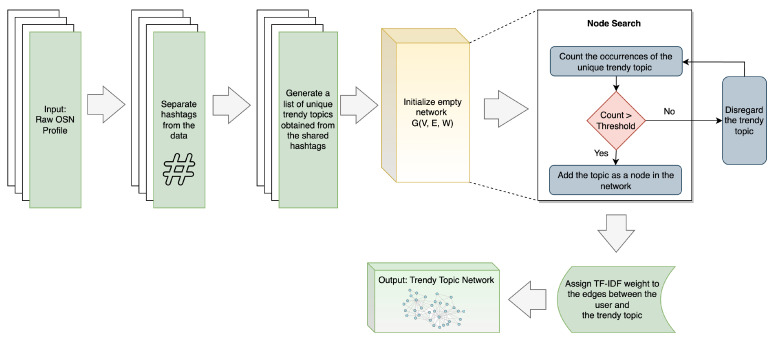
Process of generating Trendy Topic Network SBB trait, adapted from [9,44].

The Trendy Topic Network identifies the behavioral patterns of OSN users in response to popular and emerging trends in OSNs [9]. Utilizing hashtags in posts is a widespread practice in OSNs. Users frequently incorporate popular hashtags into their posts to align them with prevailing trends [9,39,44]. These hashtags reflect a user’s inclinations towards specific trends, as the primary subject matter of the posts is directly associated with the hashtags used [39,44]. The contextual information derived from this behavior gives rise to the Trendy Topic Network, also known as the Hashtag Network [9,44]. The overall workflow of generating the trendy topic network is depicted in Figure 8.

From the OSN profile, the hashtags used in posts are separated at first. Then a list of unique trendy topics is generated from the shared hashtags. To ensure the relevance and significance of the network, a threshold is set to filter out low-frequency and less influential hashtags [9,44]. Only those hashtags that meet or exceed the predetermined threshold are included in the network as nodes. Finally, a TF-IDF weight is assigned to the edges between the user and the trendy topics, using the following Equation (2) [45]:

(2) $$W_{t}=\left(1+\log\left({\mathrm{TF}}_{t,d}\right)\right)\log\left(N/{\mathrm{DF}}_{t}\right)$$

In this equation, DF refers to the document frequency, which is the number of occurrences of edge *t* across the *N* user profiles whereas TF represents the term frequency of each edge *t* within profile *d* and finally, *N* denotes the total number of user profiles [9]. The Trendy Topic Network, based on hashtag-sharing behavior in OSNs, offers a powerful tool for understanding the interests and preferences of users, as well as the dynamic nature of information sharing and trend propagation in the digital age [37,39].

### 3.6. Generation of Matching Scores

The matching score generation module, commonly found in biometric systems, calculates the similarity score for an individual. In the case of OSN users, a matcher generates the similarity score; a higher score indicates a greater likelihood of a user matching a specific user template in the biometric database [46]. The number of matchers in an SBB system depends on the number of available SBB traits. As each SBB trait generates an independent similarity score, a multimodal SBB system with *N* SBB traits will have *N* biometric matchers. In this study, a total of six biometric matchers are employed, including the human micro-expression SBB trait. To produce the matching scores, both machine learning and rule-based algorithms are typically utilized in multimodal biometric systems [9,46]. In this proposed multimodal system, the matching scores for Reply Network, Retweet Network, URL Network, and Trendy Topic Network are generated according to the techniques introduced in [9]. To generate the matching score of these four SBB traits, the method searches for nodes within the associated training network and selects a group of candidates that possess shared edges with the searched nodes [9]. Following this, the similarity score ($S_{Tr}$) between the train network ($N_{Tr}$) of each candidate and the test network ($N_{T}$) is calculated using Equation (3) [9]:

(3) $$S_{Tr}=\sum_{e\in E\left\{N_{T}\cap N_{Tr}\right\}}W_{e}$$

In this equation, $E\left\{N_{T}\cap N_{Tr}\right\}$ refers to the set of edges or relationships between the common nodes in the test network and each candidate’s train network, while $W_{e}$ signifies the corresponding weight [9]. To compute the matching scores for the writing profile, a Support Vector Machine (SVM) is employed as per [10]. For the human micro-expression trait, an ensemble of classifiers is utilized, as detailed in [11].

### 3.7. Score Fusion and Identification

The fusion of matching scores is a prevalent approach in both multimodal SBB systems and multimodal physiological biometric systems, as it enhances the overall accuracy of person identification [34,46]. In this module of the proposed multimodal SBB system, individual matchers’ matching scores are combined to generate a final matching score, which is subsequently used for user identification. The rank-level weighted Borda count technique [34], employed in the novel human Micro-expression method, is utilized for fusing the matching scores.

The rank-level weighted Borda count technique [34] is particularly well-suited for situations where the performance of different biometric matchers varies significantly [34]. Given that the standalone performance of each SBB trait differs considerably, this algorithm is chosen for fusing the matching scores, leading to improved biometric identification accuracy. In every biometric matcher, the top-ranked user identity is allocated *N* votes, while the second-ranked candidate identity receives N−1 votes, and so forth [34]. Subsequently, the votes from all biometric matchers *M* are accumulated for each potential user identity [34]. The user identity with the greatest number of votes is determined to be the actual user identity. The weighted Borda count score $m_{k}$ signifies the consensus strength among various biometric matchers [34]. Using Equation (4), the rank scores are fused [34]:

(4) $$m_{k}=\sum_{i=1}^{M}w_{i}r_{i}\left(k\right),\;k=\left\{1,2,\ldots ,N\right\}$$

where $w_{i}$ and $r_{i}$ represent the weight and ranked score attributed to the *i*th matcher, respectively. After the rank-level fusion, the method generates a decision table containing the rank-level fused scores for each user. From this decision table, the user with the highest score is chosen as the identified individual. This approach ensures that the most likely match, based on the combined information from various SBB traits, is selected. This fusion approach effectively leverages the strengths of multiple biometric matchers, making the identification process more robust and reliable. By integrating complementary SBB traits, the system can account for varying degrees of performance across individual matchers and ultimately produce a more accurate and dependable identification outcome.

## 4. Experimental Results

This section presents the experimental results of the proposed multimodal SBB system. The section is divided into five subsections where the first two describe the dataset used in the experiments and the experimental setup. Next, person identification results are analyzed for each SBB trait. Finally, the results of the proposed multimodal SBB system are discussed, followed by a comparison with the state-of-the-art.

### 4.1. Dataset

For the training and validation of the proposed SBB system, a proprietary Twitter dataset [9] is employed. The dataset is fully anonymized to protect users’ privacy, containing 250 users, with at least 200 tweets in each session per user, collected over one year and organized by four consecutive time segments [9]. The dataset not only incorporates the tweets themselves, but also includes a wealth of supplementary information such as mentions, responses, retweets, shared hashtags, shared URLs, timestamps, and location data, among others [9]. Following the extraction of relevant features, the dataset is partitioned into training and testing subsets using an 80:20 ratio, with the former allocated for model training and the latter for testing purposes. This division is carried out using a stratified sampling technique [47] to ensure a consistent representation of user data across both subsets. Importantly, the time progression of tweets is preserved during this process to maintain the temporal structure and integrity of the dataset.

### 4.2. Experimental Setup

The proposed multimodal SBB system is implemented in Python 3.9. For the machine learning pipeline, the Scikit-learn library is used. The experiments run on an Intel Xeon Silver 4210 CPU (40 Cores, 2.2 GHz) backed up with an NVIDIA Quadro RTX6000 GPU backend and 314 GB of RAM. For the experiments, a 5-fold stratified cross-validation technique [48] is employed. The hyper-parameters and the weights for rank-level fusion are optimized by using grid-search [49]. The proposed multi-modal SBB system is evaluated based on accuracy, precision, recall, and F1-score. The optimal parameters for each SBB trait are the same as originally reported in [9,10,11].

### 4.3. Results of Individual SBB Traits

In this section, an analysis of the individual Social Behavioral Biometrics (SBB) traits’ performance is conducted. According to the methods described in [9,10,28], the original retweet network, reply network, URL network, hashtag network, temporal profile, and linguistic profile for all available users are constructed. The results of person identification by standalone performance of the SBB characteristics are shown in Table 1. In the context of existing SBB methodologies, this analysis provides insightful information on the efficacy and potential of human micro-expression SBB trait to enhance multimodal SBB systems’ overall person identification performance if integrated.

The performance of the temporal profile as a social behavioral biometric is found to be the weakest, achieving a mere 9.34% accuracy in identifying individuals. On the other hand, when considering network-based SBB characteristics, the reply network outperforms others with an accuracy of 60.74%. It is crucial to highlight that for all network-based SBB traits, except for the retweet network, the F1-scores are disappointingly low compared to human micro-expression. This indicates that the human micro-expression SBB significantly outperforms the network-based and temporal SBB traits across all assessment measures.

When assessing the novel SBB feature, human micro-expression, it consistently scores higher in precision, recall, and F1-score than its traditional SBB counterparts, except for the linguistic profile. The precision score of linguistic profile is as high as 64.36%, compared to 62.14% for human micro-expression. Additionally, the linguistic profile’s recall score is around 6% higher than that of human micro-expression. However, the accuracy of human micro-expression is more than 5% lower than that of the linguistic profile. A possible explanation for the high performance of linguistic features in person identification is the considerably larger feature space generated by the linguistic profile, presented in Table 2, which consists of approximately 57,967 dimensions, due to the richness of vocabulary usage.

In contrast, the feature vector size of the human micro-expression SBB trait is only 15,000, nearly one-fourth the size of the linguistic profile’s feature space. Notably, even with a significantly smaller feature space, the accuracy, precision, recall, and F1-score of the proposed method are only slightly behind the linguistic profile. This not only emphasizes the preeminence of human micro-expression but also highlights the inherent potential of improving overall identification if integrated into a multimodal system.

Another important aspect is that due to the smaller dataset size, with only 200 tweets available for every user, the method of human micro-expression can only generate a limited number of emotion signals. This, in turn, limits the number of emotion signals to generate the samples for training, which significantly influences the learning process of the model. If the proposed method is trained on a larger dataset, it is expected to achieve better performance since the learning of the model would be much more efficient with a higher number of emotion signals being available. This suggests that the human micro-expression SBB trait would be even more efficient for person identification if trained on a larger dataset while still having a significantly smaller feature space than linguistic profile.

In addition to time efficiency, a compelling rank-wise accuracy comparison of the recently introduced human micro-expression and the linguistic profile, as depicted in Table 3, further demonstrates the superior performance of the human micro-expression SBB trait, while the linguistic profile holds a slight edge in rank-1 accuracy with 67.62% versus the human micro-expressions’s 61.73%, the human micro-expression SBB trait begins to outperform the linguistic profile from rank-2 onwards. It achieves a higher rank-2 accuracy of 77.45%, compared to the linguistic profile’s 76.23%.

This performance advantage continues to escalate as it moves further down the ranks, with the human micro-expression consistently outpacing the linguistic profile in accuracy. By rank-5, it already demonstrates a 3.19% point lead with an accuracy of 93.24%, compared to 90.05% for the linguistic profile. At the higher ranks (6 to 10), the human micro-expression SBB trait not only maintains but also extends its lead, culminating in a perfect accuracy score of 100% at ranks 9 and 10. In contrast, the linguistic profile only reaches this perfect accuracy at rank-10. Despite a slightly slower start at rank-1, the human micro-expression SBB trait swiftly surpasses the linguistic profile in accuracy from rank-2 onwards, maintaining this superiority consistently across all subsequent ranks.

**Figure 9:**
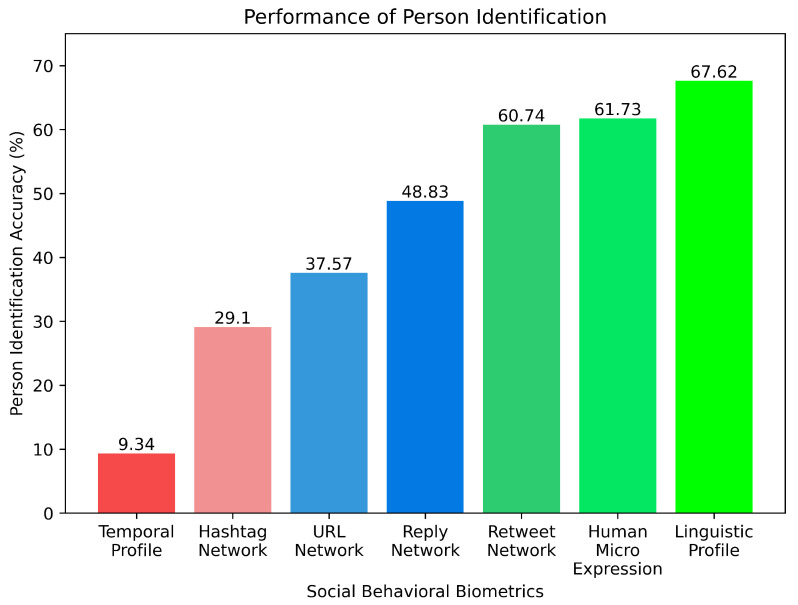
Performance comparison of different state-of-the-art social behavioral biometrics.

As depicted in Figure 9, the human micro-expression shows an identification accuracy that is over 12% greater than that of the other network-based SBB traits, except Retweet Network. Temporal profile and hashtag network exhibit poor performance, with identification accuracies below 30%. URL network and reply network fall within a similar range, achieving accuracies between 30% and 50%. Retweet network, human micro-expression, and linguistic profile SBB traits demonstrate closer proximity in terms of rank-1 accuracy, ranging from 60% to 70%.

The in-depth analysis presented above reveals that the human micro-expression SBB trait excels in both accuracy and efficiency. Its performance surpasses the linguistic profile when it comes to rank-2 accuracy and higher, and it does so while utilizing a much smaller feature space. These findings imply that the human micro-expression SBB trait holds considerable potential for incorporation into a multimodal SBB system, improving the overall identification accuracy.

### 4.4. Performance Analysis of Proposed Multimodal SBB System

This section presents a detailed analysis of the results of the proposed multimodal SBB system. The performance of the proposed multimodal system with different sets of features is examined to determine whether the novel SBB trait, human micro-expression, can be integrated with other SBB traits to contribute to improved person identification accuracy. Subsequently, a comparative analysis of the proposed multimodal system with state-of-the-art multimodal systems is provided.

To determine the potential of human micro-expression to improve person identification accuracy when integrated with other SBB traits, experiments were designed and carried out in four steps. First, a baseline feature set consisting of network-based SBB traits, namely URL network, retweet network, hashtag network, and reply network, is considered to observe the performance of person identification. Next, the human micro-expression SBB trait is integrated with the baseline SBB traits, and the impact on person identification accuracy is examined. The integration of the human micro-expression biometric is carried out with different emotion signal lengths. Since the language-based SBB trait, linguistic profile, performs better as a standalone trait, it is also added to the baseline features to understand the difference in performance improvement. Finally, the human micro-expression SBB trait is integrated with all the state-of-the-art SBB traits except the temporal profile (as it has the lowest performance that does not affect the overall system) to prove the hypothesis that human micro-expression SBB characteristic can improve person identification accuracy in a multimodal SBB system architecture. The experimental results on the proposed multimodal SBB system are provided in Table 4. The features of the URL network, retweet network, hashtag network, and reply network are termed baseline features. The integration of human micro-expression with baseline features is annotated as BH. The baseline features and the linguistic profile are called BL. Finally, the incorporation of human micro-expression with BL features, where the emotion signal length is 50 [11], is termed BLH50.

From Table 4, the person identification accuracy reaches 63.47% when only the baseline SBB traits (URL network, retweet network, hashtag network, and reply network) are considered. Compared to the highest standalone performance of baseline SBB traits, which is approximately 60.74% achieved by the retweet network, this represents a 2.73% improvement when these features are combined.

Upon integrating the human micro-expression with the baseline SBB traits, the overall person identification accuracy sees a noticeable improvement. With a signal length of 50, the improvement peaks at around 4%. With a signal length of 75, the improvement is at its lowest, less than one percent. However, the performance improves at a signal length of 25, and the maximum improvement is observed at a signal length of 50. An approximate 3% improvement in person identification accuracy is observed when the signal length is 25. The most significant improvement is found, however, in the precision scores. When transitioning from the baseline features to BH (integration of human micro-expression with baseline features), there is a 7% precision score improvement from 61% to 68%, given that the signal length is 50. Additionally, there is a 4% improvement in the F1-score.

Excluding the human micro-expression and integrating the linguistic profile in the feature set shows performance improvement since the standalone performance of linguistic profile for person identification is also high. In fact, higher than the human micro-expression. Integrating the linguistic profile with baseline SBB traits results in about 6% improvement. However, the performance improvement in precision score, 7%, is as same as BH. Both in recall and F1 score, the BL feature set improves the performance by 1%.

Finally, the human micro-expression SBB trait is integrated with both the linguistic features and the baseline features. Once the human micro-expression is added to the BL (baseline features and linguistic profile), the overall accuracy of person identification in the proposed multimodal SBB system is significantly enhanced to 73.87%. This represents a 10.4% improvement from the baseline SBB traits, up to a 6.53% improvement from BH, and finally up to a 3.97% improvement from BL with a signal length of 50. In BLH (integration of human micro-expression to BL), when the signal length is 25 and 75, a performance difference (1.23%) is observed. Interestingly, this difference is smaller than that of the BH with signal lengths of 25 and 75. One possible reason for this could be the presence of linguistic features, which are already high-performing standalone features, whereas in BH there are no linguistic features involved. The model’s performance may be influenced by the inherent ambiguity and multifaceted nature of human language, potentially leading to uncertainties when deciphering intricate textual nuances or contexts. The precision score no longer improves beyond 70% when signal lengths are 25 and 50, respectively. However, the recall score reaches its highest at 74%. The overall balance score of precision and recall is 72% as measured by the F1-score. Overall, with signal lengths of 25, 50, and 75, the BLH feature set identifies individuals with 71.56%, 73.87%, and 70.33% accuracy, respectively. Therefore, the BLH50 feature set, which incorporates the baseline features, linguistic profile, and the human micro-expression with a signal length of 50, is found to be the most optimum choice for maximizing person identification performance in the proposed multimodal SBB system.

**Figure 10:**
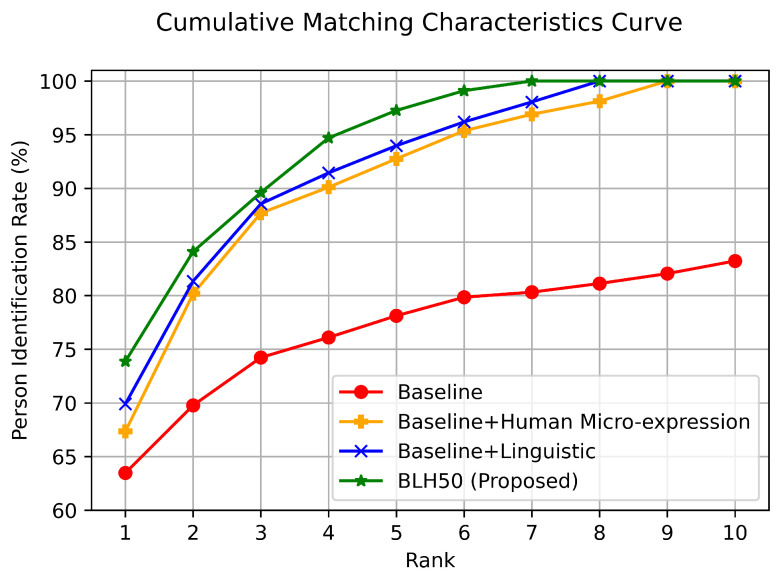
Rank-level person identification rate of the proposed multimodal SBB system.

A comprehensive understanding of the performance in rank-level person identification with these optimum feature-set settings is further analyzed through the cumulative matching characteristic (CMC) curve depicted in Figure 10. It is observed that although the person identification accuracy starts at 63.47% and 67.34% with baseline and BH feature sets, respectively, the identification rate becomes significantly higher over different ranks when the human micro-expression trait is incorporated with the baseline SBB traits. With baseline features, the person identification rate increases approximately by 6.28% at rank 2 from 63.47% to 69.77%, whereas with integrating human micro-expression, the identification rate at rank 2 has about 13% increase from rank 1. Interestingly, when the human micro-expression SBB trait is integrated, the identification accuracy becomes saturated at rank 9. On the other hand, the person identification accuracy does not become saturated within rank 10; the maximum identification accuracy at rank 10 with the baseline features is approximately 84%.

When linguistic profile is integrated with baseline features, it does not outperform the human micro-expression integrated with baseline features at rank 2. The identification improvement with BL features at rank 2 is about 12%, whereas it is about 13% with BH features. However, the identification rate with the BL feature in the subsequent ranks becomes better than with BH, as the BL reaches 100% accuracy at rank 7. Now, integrating the human micro-expression on top of BL features produces significantly better results at different ranks.

Within the first four ranks, the BLH50 feature set can accurately identify individuals 94.70% of the time, while it is 91.44% with the BL feature set. Within ranks 5 through 7, the proposed BLH50 feature set produces an accuracy score of 97.26% to 100%. As demonstrated in Figure 10, the BLH50 feature set improves the person identification rate at every rank from rank 1 to rank 10. For most feature sets, the biggest improvement in person identification takes place from rank 1 to rank 3. With BL and BH, the identification rate demonstrates a flat difference from ranks 3 through 7. On the other hand, the identification rate increases higher and then decreases lower with BLH50 from rank 3 to rank 6. Overall, the CMC curve demonstrates consecutively higher performance improvement at different ranks when the human micro-expression is incorporated. This indicates that the inclusion of human micro-expression enhances person identification across multiple ranks.

Through the extensive analysis conducted above, it is evident that incorporating human micro-expression with other SBB traits substantially enhances person identification accuracy. In each rank, the human micro-expression SBB trait demonstrates a marked improvement in person identification when compared to the majority of other SBB traits when integrated. This indicates that the integration of human micro-expression with other SBB traits, including the linguistic profile, can yield significant improvements in person identification accuracy, underlining the effectiveness and potential of human micro-expression as a valuable addition to the existing SBB traits in creating a more robust and accurate person identification system.

### 4.5. Performance Comparison with the State of the Art

This section offers a comparative performance analysis of the proposed multimodal SBB system with state-of-the-art multimodal SBB systems. To ensure a fair comparison, all state-of-the-art multimodal SBB approaches are re-implemented following the same experimental setup used for the proposed system. The multimodal approach in [9] combines the baseline features with the temporal profile. The multimodal SBB systems presented in [44] incorporate linguistic profile and stylometry features using weighted sum rule techniques, in addition to the baseline features, to enhance overall performance. Since the state-of-the-art multi-modal systems utilize score-level fusion, the proposed method has also experimented with score-level fusion alongside rank-level fusion. The comparative performance differentials yielded by these two fusion techniques are illustrated in Table 5.

This comparison reveals a negligible variance in overall performance regardless of the fusion technique implemented. The accuracy of the proposed system is 73.84% and 73.87% when score-level and rank-level fusion are utilized, respectively. Therefore, the data suggest that the enhanced performance of the method is not derived primarily from the fusion technique chosen. Instead, it is largely attributed to the prominent role of human micro-expression SBB trait, which illustrates its considerable feature importance within the models.

Now, Table 6 demonstrates the comparative results of the existing SBB systems and the proposed multimodal SBB system, showcasing the effectiveness of the proposed approach in relation to other methods.

It is noticeable that the integration of the temporal profile with the baseline features yields the same experimental results as the baseline features presented in Table 4. Given the low standalone person identification accuracy (below 10%) of the temporal profile based on the experimental setup used in this study, the weight of the temporal profile is minimal, which does not contribute to the overall performance improvement. The multimodal SBB system in [44,50] achieves an accuracy of 70.59%, while the precision score is relatively low at 66%, the recall score is 60% with an F1 score of 68%. This represents a 7% improvement over the results from [9]. The updated SBB system incorporating stylometry features in written samples [44] further improves identification accuracy by around 1%. Interestingly, precision and F1 scores increase by 4% and 2% respectively.

With the proposed multimodal system that integrates the human micro-expression SBB trait alongside the baseline and linguistic features, there is an approximate increase of 3% in person identification accuracy. The F1 score also improves to 72%, representing a 4% increase. The lowest performing multimodal SBB approach is the incorporation of baseline and temporal profile, with an accuracy score of 63.47%. The second-highest performing multimodal SBB system is [44,50], achieving an accuracy score of 71.38%. The proposed multimodal SBB system, which integrates the human micro-expression SBB trait with baseline and linguistic features and utilizes the rank-level weighted Borda count technique, outperforms the state-of-the-art multimodal SBB systems with an impressive accuracy score of 73.87%. Although the obtained result is not perfect, it is crucial to note that behavioral-based biometrics tend to be less precise than physiological biometrics [4,51]. Given that this approach is centered on human communication, the results show promise. Importantly, the state-of-the-art SBB traits and mulitmodal systems originally required at least 200 tweets to identify a person. On the other hand, the proposed multimodal system integrating human micro-expression requires only 100 tweets to identify a person, which is a significantly lower data requirement than the state-of-the-art. This highlights the significant potential and effectiveness of the novel human micro-expression SBB trait in enhancing person identification performance when integrated with other SBB traits.

## 5. Conclusions

This paper introduced a novel multimodal SBB system, focusing on the integration of human micro-expressions from text with other established SBB traits for online user identification. The fusion of these SBB traits has proven to be instrumental in improving user recognition rates, hence affirming the significance and compatibility of the novel human micro-expression biometric trait within a multimodal framework. From an OSN user’s profile, the proposed method extracts six different SBB traits and integrates them into a multimodal SBB system to identify who that person is. Experimental results demonstrate that the integration of human micro-expression SBB trait with the established SBB features within the proposed system led to a notable increase in person identification accuracy, yielding approximately a 10% improvement when only the network-based SBB traits are considered without human micro-expression SBB trait; and approximately an overall 3% increase compared to the best performing state-of-the-art multimodal SBB system. Moreover, the proposed multimodal system requires only 50% of the data input when compared to existing methods to identify a person, thus improving the efficiency of the existing methods substantially. These results suggest that the human micro-expression SBB trait, defined as subtle textual cues indicating emotional states or reactions, can be incorporated with other SBB traits to enhance the performance of SBB systems for robust online user identification. The proposed system can be employed to make users’ online experiences safer by providing an additional layer of continuous user authentication. By identifying users based on their unique social behavioral traits, the risks associated with identity theft and unauthorized access can be reduced. The current methodology in generating human micro-expressions within the multimodal SBB system has room for advancement. One major limitation is the use of only six emotion classes, which can restrict the granularity of the resulting feature space. A more detailed emotion feature map can be explored to achieve a richer human micro-expression feature space to further improve the performance of the proposed multimodal SBB system. Transformer-based deep learning models can be utilized for more accurate emotion recognition from text within the human micro-expression SBB trait workflow. Furthermore, the proposed system utilizes a rank-level ensemble modeling technique which contributes to slower inference capability. More efficient feature-level or decision-level optimization techniques can be investigated to overcome this limitation. Finally, deep learning techniques can be explored within the identification pipeline using a much larger dataset to investigate the scalability of the proposed method.

## Figures and Tables

**Table 1:** Person identification performance of individual SBB traits.

SBB Trait	Precision	Recall	F1-Score	Accuracy
URL Network	34.85%	37.23%	36.00%	37.57%
Retweet Network	57.09%	58.39%	57.73%	60.74%
Hashtag Network	19.46%	26.65%	22.49%	29.10%
Temporal Profile	6.39%	10.05%	7.81%	9.34%
Reply Network	46.19%	47.67%	46.92%	48.83%
Linguistic Profile	64.36%	67.55%	65.92%	67.62%
**Human** **Micro-expression**	**62.14%**	**61.32%**	**61.70%**	**61.73%**

**Table 2:** Feature vector size comparison of human micro-expression and linguistic profile.

SBB Trait	Feature Vector Size
Linguistic Profile	57,967
**Human Micro-expression**	**15,000**

**Table 3:** Rank-wise performance comparison of human micro-expression and linguistic profile.

SBB Trait	Rank-1	Rank-2	Rank-3	Rank-4	Rank-5	Rank-6	Rank-7	Rank-8	Rank-9	Rank-10
Linguistic Profile	67.62	76.23	81.58	85.70	90.05	93.34	95.61	97.11	98.90	100
**Human Micro-expression**	**61.73**	**77.45**	**85.40**	**90.85**	**93.24**	**95.53**	**97.68**	**99.15**	**100**	**100**

**Table 4:** Experimental results with different feature-set settings of the proposed multimodal SBB system.

SBB Traits	Signal Length	Precision	Recall	F1 Score	Accuracy
Baseline (URL + Retweet + Hashtag + Reply)	-	0.61	0.64	0.62	63.47%
Baseline + Human Micro-expression (BH)	50	0.68	0.65	0.66	67.34%
Baseline + Human Micro-expression (BH)	75	0.62	0.64	0.63	64.20%
Baseline + Human Micro-expression (BH)	25	0.64	0.65	0.64	66.52%
Baseline + Linguistic Profile (BL)	-	0.68	0.66	0.67	69.90%
BL + Human Micro-expression (BLH)	25	0.70	0.68	0.69	71.56%
BL + Human Micro-expression (BLH)	75	0.69	0.67	0.68	70.33%
**BLH50 (Proposed)**	**50**	**0.70**	**0.74**	**0.72**	**73.87%**

BLH50 = Baseline Features + Linguistic Profile + Human Micro-expression with a signal length of 50.

**Table 5:** Rank-level vs. Score-level fusion comparison of the proposed method.

Fusion	Precision	Recall	F1 Score	Accuracy
Rank-level	0.70	0.74	0.72	73.87%
Score-level	0.71	0.73	0.72	73.84%

**Table 6:** Performance comparison of the proposed multimodal SBB system with state of the art.

Multimodal SBB System	Feature Set	Precision	Recall	F1 Score	Accuracy	Year
Madeena et al. [9]	Baseline + temporal profile	0.61	0.64	0.62	63.47%	2017
Tumpa et al. [50]	BLWSR	0.66	0.70	0.68	70.59%	2022
Tumpa [44]	BLSWSR	0.70	0.70	0.70	71.38%	2022
**BLH50 (Proposed)**	**BLHWBC**	**0.70**	**0.74**	**0.72**	**73.87%**	**2023**

BLWSR = Baseline SBB traits + linguistic profile fused using weighted sum rule. BLSWSR = Baseline SBB traits + linguistic profile + stylometry fused using weighted sum rule. BLHWBC = Baseline SBB traits + linguistic profile + human micro-expression fused using weighted Borda count.

## Data Availability

The data is proprietary and unavailable due to privacy restrictions.
